# Bacterial Performance in Crack Healing and its Role in Creating Sustainable Construction

**DOI:** 10.1155/2022/6907314

**Published:** 2022-07-07

**Authors:** Digafe Alemu, Wubetie Demiss, Gamachis Korsa

**Affiliations:** ^1^Center of Excellence for Biotechnology and Bioprocess, Addis Ababa Science and Technology University, P.O. Box 16417, Addis Ababa, Ethiopia; ^2^Department of Biotechnology, College of Biological and Chemical Engineering, Addis Ababa Science and Technology University, Addis Ababa 16417, Ethiopia

## Abstract

Building practices began with human civilization. Cement is the most commonly used building construction material throughout the world. These traditional building materials have their own environmental impact during production, transportation, and construction, but also have limitations on building quality and cost. Biological construction materials are currently emerging technology to combat emissions from the construction sector. Different civil and biotechnology researchers have turned to microorganisms for the production of bio construction materials that are environmentally friendly, socially acceptable, and economically feasible but can also produce high strength. Scanning electron microscope (SEM) and X-Ray diffraction (XRD) are the most characterization methods used to observe and ensure the production of calcite precipitate as bacterial concrete. As compared to conventional concrete, bacterial concrete was greater by 35.15% in compressive strength, 24.32% in average tensile strength, and 17.24% in average flexural strength, and it was 4 times lower in water absorption and 8 times lower in acid resistivity than conventional concrete. Genetic engineering has great potential to further enhance the mechanical strength of bacterial concrete for use in crack repairs in existing buildings.

## 1. Introduction

Building construction is not a recent technology; it began with human civilization. In 3000 BC Sumerians used a mixture of clay and straw complexes as biocomposite materials [1]. Nowadays cement is the most commonly used building construction material throughout the world in the form of concrete and block [2]. To fulfill the need of housing, a total of 12 billion tons of concrete materials are produced annually, about 2 tons per person [3]. For this about 4.4 billion tons of cement was produced globally in 2021 [4]. Among all building materials, concrete attains special features in the construction activities due to its durability, high compressive strength, and resistant to chemicals and weathering actions [5].

Nowadays conventional construction practice such as material production, construction, and demolition holds a role in environmental pollution. During cement manufacturing, varying gases are released to the environment due to heating of clay and limestone. About 2.7 billion tons of carbon dioxide released from manufacturing of cement in 2021 [6]. Similarly, one recent study shows that, about 8.7 gigatons, which is about 10% of the world emission, of carbon dioxide is from construction sector [7]. Other study done by Zhao et al. [8] also pointed out that, in 2019 the total waste produced from demolition and construction in China alone accounts about 2300 million tones became the largest construction and demolition waste producer in the world. These all the above study shows that construction sector has greater contribution on environmental pollution

The other drawback of cement-based construction is crack formation. Concrete crack is widespread phenomenon in concrete-based buildings. Cracking causes a decrease in engineering properties and construction material strength in the surface layers of concrete. It often happens due to the pore size between the materials, low tensile strength of the material [9], overloading, improper design, quality of supplementary materials, and lack of skilled labor [10]. The crack that formed allows penetrating unwanted materials inside and if regular maintenance and special treatment is not done, it is causing damage to the building and leads to reinforcement corrosion and unexpected maintenance cost [11].

Studies show that, to enhance the durability and prevent penetration of aggressive materials, it can be treated through passive (exterior) and active (interior and exterior) methods [12]. External treatment consists of chemical mixtures and polymers either through sealant injection or spray. Those chemical mixtures include epoxy resins, chlorinated rubber, waxes, polyurethane, acrylics, and siloxane [13]. Whereas the active treatment method which is called “self-healing” is automatically healed crack both internally and externally through three main strategies: (i) autogenous healing, (ii) encapsulation of polymeric material, and (iii) microbial production of calcium carbonate (autonomous healing) [12]. Autogenously healing is a natural process hydration of unhydrated cement particles and can heal a narrow cracks not more than 0.3 mm, and it strongly depends on humidity availability, whereas encapsulation of polymeric material is filling cracks through released chemicals from incorporated hollow fibers [13]. The passive method negatively affects the concrete through extending the existing cracks and need extra materials like capsules. In contrast, active method mainly microbe-based method, is extremely desirable, sustainable, and ecofriendly. It also advantages in long shelf life high quality, and frequent crack healing [14]. Whereas, passive treatment techniques are low heat resistance, poor weather resistance, moisture sensitivity, poor bonding with concrete, unsustainability, susceptibility to degradation and delamination with age and thermal expansion coefficients between concrete and sealers, time consuming, and high cost [13].

## 2. Application of Microorganisms in Construction Sector and their Significance

The idea of microbial application for construction materials were first introduced in 1990 by the US research group Prof Sookie Bang, Eben Bayer, and Gavin McIntyre [15]. Microbe such as bacteria used directly or indirectly for self-healing of cracks formed on the concrete ( [9, 16, 17]; [18, 19], and as biobrick production [20, 21], while fungus are also used as myco block production through substrate mycelium complex [15, 22, 23] and crack self-healing through calcite production [24, 25]. Table. 1 shows the selected microbial species applied in the construction sector. According to Ivanov et al. [28] studies in construction microbial biotechnology, two major directions are used, (1) indirect method, through producing soil stabilizing enzyme from microbes, and (2) direct methods, direct application of microorganisms in the construction process. The unique nature of microorganisms such as metabolic process, cell wall, reproductive rate, growth rate, spore formation, and metabolite formation plays a great role for microbial diverse applications [9].

## 3. Microbial Construction Materials

Different studies have shown that varying species of bacteria and fungi have the capacity to mineral precipitation, mainly calcium carbonate, which plays a great role in concrete strengthening.

Microorganisms that can induce carbonate precipitation are: (i) sulphate reducing microorganisms, (ii) photosynthetic microorganisms, and (iii) microorganisms involved in the nitrogen cycle [26]. The main role of these microbes is to produce catalytic agents and create an alkaline environment through their metabolic activities [17]. Most microbial mediated construction materials include;

### 3.1. Bio Cementation

**Figure 1:**
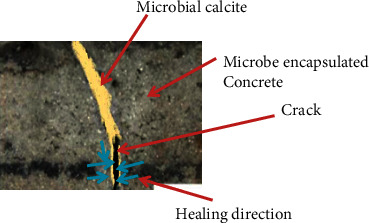
Bacterial self-healing process.

Biocementation is a process of production of carbonate precipitate (MICP) from microbes [26]. As shown in Figure 1, bacteria precipitate Calcite from available calcium sources and form biocement which is directly applied in self-healing of microcracks [9, 11, 21, 26]. Selection of bacteria is based on spore formation, harsh environment tolerance, nutrient survival, high alkalinity, growth conditions, and resistance to mechanical stress [10]. The applied microbes should withstand high alkalinity, deficient oxygen, and precipitate calcite when mixed with healing agents. Because the produced calcium carbonate change the environment more alkaline.

Studies show that, the genus *Bacillus* is most widely used species for bacteria-based crack healing [9, 20, 34, 35]. This genus is characterized as spore forming bacteria, with a thick cell walls, compact round shape and typically in the size range of 0.8–1 *μ*m [9], could survive about 50 years [11, 36], for hundreds of years [27], has the ability to withstand harsh environmental conditions like chemicals, high mechanical stresses as well as ultraviolet radiations.

Other studies show that the spores of *bacillus spp*. can remain dormant up to 200 years in harsh environment [5]. When cracks occur, the capsules are ruptured and the healing agents are released, these dormant bacteria spores easily gets moisture from the air and triggers the cell to germinate, grow and form calcite and seal the cracks in situ [11, 21, 36, 37]. Due to the dissolving of calcium hydroxide obtained from concrete with available moisture, the pH of the environment rises from 6.5 to 13 [30, 33]. Then the available bacteria prepare themselves to withstand the alkaline environment.

The day taken to accomplish the crack healing mainly depends on the length of width of the crack and the number of curing days. Most bacterial strains are efficient in crack healing with crack widths 0.15 mm [36]; 0.2 mm; 0.5 mm [9, 16, 38]; 0.8 mm [33]; and 1 mm [11], and with the curing time of three week [21], three to four weeks [37] and three to fourteen weeks [11]. The content of water inside the crack becomes reduced as the cracks are completely healed, as a result the bacteria will become dormant again [11]. Even though the technology is environmentally friend and economically feasible, further studies needs to be done to improve the healing width and the lifetime of the bacteria through genetic modification.

Most extra ingredients for self-healing concrete include bacterial strains, hydrogels, and different carbon sources such as manganese, peptone, calcium glutamate, and calcium lactate [27]; corn step liqueur and sugar cane meal [26]; and urea [16]. Added carbon sources are the primary source of nutrients for bacteria in the produced concrete, and finally they become dormant for a long period of time as the nutrients are depleted. Self-healing of cracks is not efficient by the addition of bacteria alone; it is more prominent when calcium lactate is used as a biomineral precursor [39, 40]. To solve the dehydration problem hydrogel acts as a water tank for the continuous crack healing process [16]. Hydrogel can also help the bacteria resist the mechanical force. Bacteria spores incorporated in the hydrogel can fill a wider diameter of the crack than the spore without the hydrogel [16]. Organic compounds such as peptone and calcium glutamate are used as energy and carbon sources for growth [40].

### 3.2. Bio Brick

Biobricks can be produced through calcite precipitation of alkaline resistant spores forming microbes, bacterial and fungus metabolism as well as chemical reactions [20, 41].

### 3.3. Biopolymer

Biopolymers are polysaccharides produced by microorganisms such as bacteria, algae, and fungi as a result of metabolic activity. Chang et al. [29] concluded in their study that biopolymers such as xanthan gum produced by *Xanthomonas campestris* and gellan gum produced from *Sphingomonas elodea* are used to replace cement for soil strengthening. Their findings show that soil containing 0.5% (5 Kg) biopolymer resulted in a higher compression strength than that of 10% (100 kg) soil without biopolymer which is about 6.31 MPa. Soil mixed with biopolymer has twice the adhesive capacity of ordinary cement [29]. Using biopolymer as a construction material can decrease the environmental impact and has a lower cost [42].

### 3.4. Production Mechanism

There are four methods in which bacteria are mixed with other healing agents: (i) by direct method, bacteria and other agents are mixed with water to produce calcite [13, 43]; (ii) vascular network method, injection of bacteria into the concrete matrix through a vascular network system like human bone structure [13]. The embedded vascular network during concrete preparation breaks and bacteria move towards the crack position and start growth. (iii) the encapsulation method [21, 43] and (iv) the protection method; in which bacteria are incorporated within a coating material and expanded clay, respectively [21].

**Figure 2:**
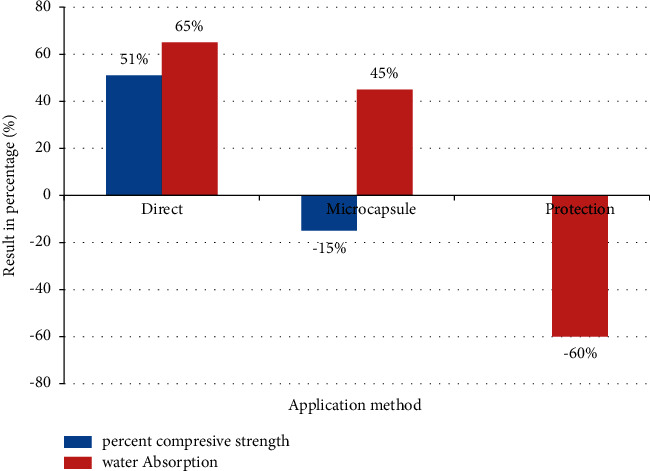
Compressive strength and water absorption ability of cement mortar made of *Bacillus sphaericus* with varying application methods [21].

The third method is the most effective method for supplying the spore with long-term effects [5, 43]. In contrast, the protection method affects the homogeneity of the concrete through weakening between the concrete compositions [21]. Each method plays a great role in the strength of concrete.  Figure 2 shows the comparative utilization of each method.

From the figure above, direct application of *Bacillus sphaericus* to the cement mortar enhances the compressive strength by 51 percent and decreases water absorption by 65 percent, which is lower than microcapsule method. Whereas the compressive strength reduced by 15 and 60 percent in microcapsule and protection method, respectively. The microbial product is characterized through: scanning electron microscopy (SEM) to visualize the precipitates; X-ray diffraction (XRD) to confirm the production of calcite; thermo-gravimetric analysis (TGA) to determine the moisture, ash, and volatile matter; compressive strength and tensile strength testing to test strength and tensile of the product [44].

Study done by Jin et al. [11] and Menon et al. [33] point out that bacteria can precipitate calcite (calcium carbonate) through three different pathways: (1) hydrolysis of urea to ammonia via the enzyme urease in calcium-rich environment; (2) conversion of organic compound to calcite by metabolic process, and (3) dissimulatory nitrate reduction. This process can be governed by the following four key factors [17]:

- the concentration of calcium sources,
- pH
- dissolved inorganic carbon concentration (DIC), and
- The availability of nucleation sites

#### 3.4.1. Urea Hydrolysis

This process is the simplest pathway for microbe-based calcite precipitation as part of metabolism [17]. Urease enzymes obtained from bacteria hydrolyzes one mole of urea to produce one mole of ammonia and one mole of carbonates (equation (1)). Spontaneous carbonates are hydrolyzed to form an additional 1 mole of ammonia and carbonic acid (equation (2)) which results in the increasing alkalinity of the surrounding environment [17]. The net increase in pH is due to ammonium and hydroxyl ion. The hydroxyl ions obtained from ammonia hydrolysis exceed the calcium ion available for biomineralization (equation (3)) [19]. The rise of alkalinity helps in facilitating the transformation of carbon dioxide to carbonate [13].

(1) $$\begin{array}{c} \text{CO}{\left({\text{NH}}_{2}\right)}_{2}+{\text{H}}_{2}\text{O}⟶{\text{NH}}_{2}\text{COOH}+{\text{NH}}_{3} \end{array}$$

(2) $$\begin{array}{c} {\text{NH}}_{2}\text{COOH}+{\text{H}}_{2}\text{O}⟶{\text{NH}}_{3}+{\text{H}}_{2}{\text{CO}}_{3} \end{array}$$

(3) $$\begin{array}{c} 2{\text{NH}}^{3}+2{\text{H}}_{2}\text{O}⟶2{\text{NH}}^{4+}+2{\text{OH}}^{-} \end{array}$$

According to the following equationstated in Seifan et al. [13]; reactions of hydroxide ions obtained from ammonia hydrolysis with carbonic acid produce carbonate (CO_3_^2−^).

(4) $$\begin{array}{c} {2\text{OH}}^{-}{\;+\text{ H}}_{2}{\text{CO}}_{3}\;⟶{\text{CO}}_{3}^{2-}{+\;2\text{H}}_{2}\text{O} \end{array}$$

**Figure 3:**
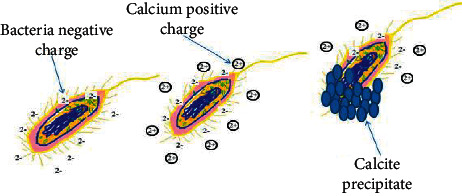
Mechanism of calcite production through cell and calcium ion attraction adopted from [45, 46].

As illustrated in Figure 3 and equations (5) and (6), an attraction force between calcium ions provided from different calcium sources and the bacterial cell wall holds each other due to the opposite charge between the calcium elements and the negative charge cell wall due to the presence of teichoic acid linked to peptidoglycan [13, 17–19], and other anionic negatively charged groups such as alcohol, amine, carboxyl, ester, phosporyl, sulfonate, hydroxyl, sulfhdryl, thiol, and thioether [47].

(5) $$\begin{array}{c} {\text{Ca}}^{2+}+\text{Cell}⟶\text{Cell}-{\text{Ca}}^{2+} \end{array}$$

(6) $$\begin{array}{c} \text{Cell}-{\text{Ca}}^{2+}+{\text{CO}}_{3}^{2-}⟶\text{Cell}-{\text{CaCO}}_{3} \end{array}$$

However, this process of calcium carbonate precipitation has some drawbacks in nitrogen oxide emission into the atmosphere through production of ammonium ions (NH^4+^) through the ureolytic activity [13].

#### 3.4.2. Metabolic Process

Metabolic conversion of organic compounds to calcium carbonate has been proposed to address the drawbacks of ureolytic hydrolysis. Seifan et al. [13] point out in their study that aerobic oxidation of calcium lactate leads to the production of sustainable biominerals in an alkaline environment by bacteria. Oxygen, moisture, and calcium lactate inside the concrete are utilized by bacteria to carry out their metabolic activity, which results in the formation of calcium carbonate as in the following equation:

(7) $$\begin{array}{c} \text{Ca}{\left({\text{C}}_{3}{\text{H}}_{5}{\text{O}}_{2}\right)}_{2}+{7\text{O}}_{2}\text{metabolic conversion}⟶{\text{CaCO}}_{3}+{5\text{CO}}_{2}+{5\text{H}}_{2}\text{O}. \end{array}$$

In addition, calcium carbonate was also obtained by the reaction of carbon dioxide with calcium hydroxide. The CO_2_ obtained from bacterial respiration can react with the calcium hydroxide ((Ca(OH)_2_)) present in the cement, producing even more calcite precipitate as shown in the following equation[9, 36]. Because of the active metabolic conversions of calcium nutrients and bacteria present in the concrete, this process is more efficient [48].

(8) $$\begin{array}{c} {\text{CO}}_{2}+\text{Ca}{\left(\text{OH}\right)}_{2}⟶{\text{CaCO}}_{3}+{\text{H}}_{2}\text{O} \end{array}$$

Compared to the ureolysis pathway, metabolic conversion is more sustainable due to the absence of emission from ammonium production through hydrolysis of urea [13].

#### 3.4.3. Nitrate Reduction

It is a respiratory process that results in the reduction of nitrate (NO^3−^) to different oxides of nitrogen (i.e., nitrite (NO_2_^−^), nitrous oxide (N_2_O), nitric oxide (NO), and nitrogen gas (N_2_)). This is another pathway to produce biology-based calcium carbonate precipitates [11]. According to equations (9)–(11) stated in [13], denitrifying bacteria such as *Diaphorobacter nitroreducens* and *Bacillus sphaericus* which are mentioned in (Table 1) result in calcium carbonate precipitation through oxidation of organic compounds by the reduction of nitrate (NO_3_^−^). The primary results of organic compound denitrification are carbon dioxide, water, and nitrogen (equation (9)). Due to the consumption of hydrogen ion, the pH become raised and form carbonate or bicarbonate production (equation (10)). Finally, the reaction of calcium source with carbonates produces the precipitation of calcium carbonate (equation (11))

(9) $$\begin{array}{c} \text{Organic compound}+a{\text{NO}}_{3}+\;e{\text{H}}^{+}\text{Denitrification}⟶i{\text{CO}}_{2}+o{\text{H}}_{2}\text{O}+u{\text{N}}_{2}, \end{array}$$

(where *a*,*e*,*i*,*o*,*u* are coefficients of the equation)

(10) $$\begin{array}{c} {\text{CO}}_{2}+{2\text{OH}}^{-}⟶{\text{CO}}_{3}^{2-}+{\text{H}}_{2}\text{O} \end{array}$$

(11) $$\begin{array}{c} {\text{Ca}}^{2+}+{\text{CO}}_{3}^{2-}⟶{\text{CaCO}}_{3} \end{array}$$

However, different factors can accelerate or decelerate the biomineralization of calcium carbonate. Selection of low risk bacteria with high ability of calcium carbonate precipitation, optimum amount of nutrients, enzyme activity and growth rate, and inoculum size are preferred for maximal precipitation [13]. Among the above-mentioned pathways, calcium carbonate precipitation through ureolysis is the fastest pathway [13], and dissimilatory nitrate reduction takes more time than the other two pathways [30, 33].

## 4. The Significance of Bacteria-Based Construction Materials

### 4.1. Cost

**Figure 4:**
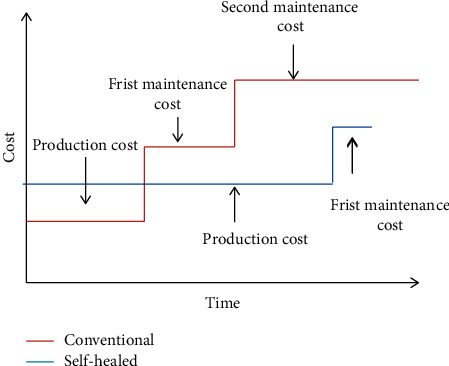
The cost analysis estimate of self-healed concrete (blue line) and conventional concrete (red line), adopted from [50].

Different studies show that the budget allocated for concrete crack repair and maintenance is more than for concrete production. Silva et al. [49] cost comparative studies on crack repair and concrete production showed that the cost of crack repair and maintenance has been estimated at $147/m3, while that of concrete production cost ranges between $65 to $80/m^3^. A similar study done by Sidiq et al. [50]. The cost of production is about 10 times higher than the cost of maintenance and repair. This shows that the cost allocated for crack repair and maintenance is twice as high as the production. Therefore, the application of bacterial self-healing technology will minimize the large investment of maintenance, labor, and repair costs through the delivery of more sustainable and cost-effective structures. Even though bio-based concrete may have a slight increase in initial cost, it significantly decreases repair and replacement costs over the lifetime of a building and it would enable the technology to be used for a wide variety of structures [51]; the economic benefit can be potentially greater over the life cycle ([50]).  Figure 4 shows the relationship between cost and life cycle.

### 4.2. Sustainability

Autogenously and encapsulation self-healing mechanisms are automatic healing cracks without human intervention or interference. During the encapsulation of polymeric material, the filler released from hollow fibers embedded in the concrete matrix is not usually compatible with concrete compositions, which could cause the enlargement and propagation of the existing cracks [11]. It needs continuous follow-up, additional cost, healing not more than 0.3 mm, a complex process, and the released chemicals have their own impact on the quality of the healed crack.

However, the bacteria-based self-healing approach can reduce all drawbacks observed in autogenous and encapsulation methods. There is no need of filler (chemicals). Instead, it uses microbe-based mineralization. Successful implementation of the biological approach treatment methods will result in a longer lifespan of concrete structures, low cost, and increased environmental sustainability, as well as significant reduction in cement production and structural replacement [13]. Furthermore, the increased longevity of self-healing concrete would result in a decrease in cement production, which indirectly leads to minimized carbon dioxide emissions [51]. As a result, it has the capacity to reduce carbon emissions nearly 800 million tons per year [20].

### 4.3. Strength

#### 4.3.1. Compressive Strength Test

**Figure 5:**
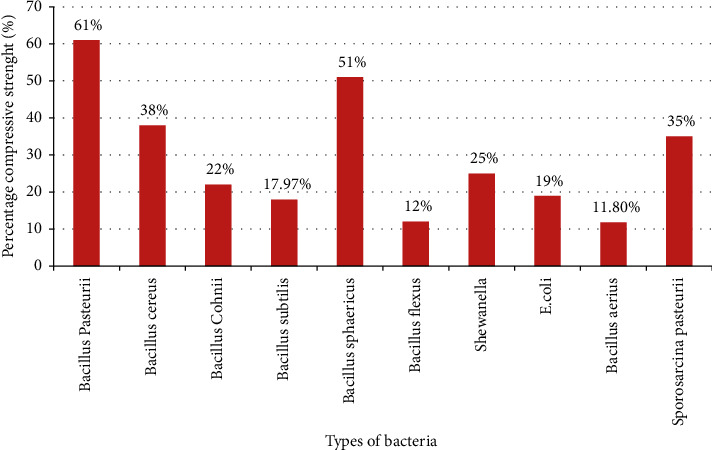
Compressive strength of mortar with different bacterial species by 10^5^ cell/ml concentration [21].

Compressive strength mainly depends on factors such as type of bacteria, concentration of bacteria, application method [21], and number of days of incubation [35]. As Ponraj et al. [35] concluded, the effects on compressive strength fluctuate between 10 to 30% as the types of bacteria are varied. Study done by Iheanyichukwu et al. [43] also stated that, by using 10^5^ cell/ml concentrations of bacterial species *Sporosarcina pasteurii* species show better compressive strength of concrete followed by *Bacillus aerius* as shown in Figure 5. The two authors, Kumarappan and Sudharsan [20], conclude that the compressive strength of biobrick is about 10 Mpa [52], which is 19% higher than conventional brick and 3 times lighter in weight than conventional brick after 28 curing days of experiment.

The addition of more number of bacteria negatively affects the quality of concrete [53]. This is because as the density of cells increases, there is high nutritional competition with each other. When the concentration of bacteria cells increased more than the optimum level, it reduces about 10% of the compressive strength compared to conventional concrete [27]. Stanaszek-Tomal [53] concludes that 10^5^ cell/ml bacteria concentration with 28 curing days shows better compressive strength.

According to Borse et al. [34] experiment, from 5, 10, and 15 milliliters of bacterial concentration 32.65, 33.06, and 34.53 Mpa is reported, respectively, at 28 days which is higher value compared to conventional concrete, having compressive strength of 20.90, 26.15, and 31.65 Mpa. A similar study done by Magil et al. [37] also show that, out of 10^5^ cell/ml concentration of bacteria for 7, 14, and 28 curing days results in 14.89, 16.42, and 19.26 Mpa compressive strength, respectively, which is higher than conventional concrete, having a compressive strength of 10.03, 11.38, and 12.49 Mpa as shown in Table 2.

#### 4.3.2. Tensile and Flexural Strength Test

Tensile strength in the case of a concrete specimen is defined as the stress developed by the application of a load due to compression at which the concrete specimen may crack. The result illustrated in Table 2 shows that at 10^5^ cell/ml bacterial concentration, the tensile and flexural strength increase with increasing number of curing days [37]. 28 curing days is optimum for both tensile and flexural strength having a value of 19.26 and 10.33 Mpa, respectively. The length of curing days helps the bacteria to colonize the agents and produce efficient enzymes. An experimental study done by Borse et al. [34] also showed that, from 5, 10, and 15 ml of cell concentration with 7, 14, and 28 curing days, 15 ml of cells with 28 curing days resulted in the maximum flexural strength. Compared to conventional concrete, bacterial concrete increases 35.15% in compressive strength, 24.32% in average tensile strength, and 17.24% in average flexural strength [37].

#### 4.3.3. Durability and Water Absorption Test

The other advantages of bacteria-based construction materials are resistance to acids and less water absorption. It can decrease water absorption by four times [16], six times [17], and eight times, decrease chloride permeability by 45%, decrease the porosity of mortar specimens, [16] increase electrical resistance, and decrease electrical charge passing through it [54]. This is because of microorganisms precipitating calcite and leading to a minimized void and hence a lesser permeability [29]. According to [16, 37] studies, bacterial concrete has less percentage strength loss than conventional concrete immersed with 5% H_2_SO_4_. They conclude that conventional concrete at 36.5 N/mm^2^ reference age and bacterial concrete at 46.4 N/mm^2^ reference age at 28 curing days, only 10.7% strength is loosed from bacterial concrete and 16.4% from conventional concrete. As the number of curing days increases, the durability decreases in both conventional and bacterial concrete at a constant concentration and type of acid. However, the rate of loss of strength is higher in conventional concrete than in bacterial.

## 5. Limitations of the Technology and its Future Trends

There are some drawbacks to biologically mediated crack self-healing technology such as spore dormancy period, narrow crack repair, loss of concrete compressive strength due to incorporated capsule [11]. Even though bacteria have a fast growth period, they are sensitive to harsh environmental conditions such as high alkalinity (pH up to 13), high temperatures, and limited oxygen supply compared to fungi [11, 51]. It is difficult for most bacterial strains to survive in harmful environments. It is due to the fact that the size of the spore is larger than the pore size in concrete and to increase the survival period, encapsulation is needed to save the spore from damage during the hydration process. However, encapsulation may affect the concrete's compressive strength. Bacterial cell wall's surface area to volume ratio is smaller than that of fungus. This nature of the bacterial cell wall limits the calcium mineral precipitation. It is possible to enhance the bacterial life span and extracellular polymeric substances through genetic engineering to result in better self-healing [51, 55, 56]. To increase bio-based self-healing technologies on the market, inter-and intra-gene modification needs to be considered. Intragene modification of alkalophilic and thermophilic bacteria needs to be accelerated. Intergene transformation is gene transformation in different species. Bacteria with high calcium producing ability can translocate the gene to spore forming and harsh environmental resistant fungi, typically filamentous fungi. This will combat the drawbacks observed in bacteria-based concrete.

### 5.1. The role of Gene Transformation in Bacteria-Based Self-Healing Concrete

Short lifetime is one of the limitations of bacteria inside the concrete matrix which restricts it from behaving as a true self-healing activator over sustained duration. It is possible to enhance the bacterial survival period in the harsh environment through genetic transformation of alkaliphilic bacteria and thermophilic anaerobic bacteria. A study done by Sarkar et al. [55] concluded that a genetically modified *E. coli* bacteria (BKH2) through bioremediase-like protein incorporation was increasing the mechanical strength and durability of the mortar samples due to its capacity to leach silica from silicate substrates. However, this modified gene lacks the ability to adapt to an alkaline environment. In contrast to this, most *Bacillus* species can survive in an alkaline environment and form large spores. Studies done by Sarkar et al. [56] showed that biotransformation of a biosilification genes (bioremediase-like protein genes) from the BKH2 strain to the spore forming bacteria *Bacillus subtilis* (*B. subtilis*) through T-vector can increase the mechanical strength of the concrete, and the new modified gene has the capability to live a long life span, produce more calcite, and have a high self-healing capacity. A similar study was also done by [57].

Similar studies done by Lee and Park [51] revealed that bacterial gene transformation is used to increase bacterial extracellular polymeric substances and express green fluorescent protein to easily observe the MICP. Most of the time, precipitated calcite is observed through SEM, but it is also possible to produce green fluorescent protein through gene transfection of *Pseudomonas aeruginosa* MJK1 and *Escherichia coli* MJK2 to study MICP [51]. This transfected gene enables the observation of MICP processes in model systems such as mortar specimens. In addition, urease genes from *Sporosarcina pasteurii* were transfected to bacteria naturally capable of producing large amounts of extracellular polymeric substances, *Pseudomonas aeruginosa* strains 8821 and PAO1, to get new genes with high alkaline adaptive capacity and good calcite precipitator [51]. The new gene can survive in a high alkaline environment and produce an extracellular polymeric substance. Generally, bacterial gene transformation helps bacterial cells to mediate accelerated CaCO_3_ precipitation for sealing wide cracks. However, attention should be paid during the release of the new gene into the environment.

## 6. Conclusion

Building construction is not a new technology. It has begun since human civilization. However, the design and construction materials become vary. Currently, cement is the most widely used construction material in the form of concrete or blocks. Besides its high compressive strength and low water absorption, cement has its own social, economic, and environmental problems. About 2.7 billion tons of carbon dioxide will be released from the manufacturing of cement in 2021. If no action is taken on the use of cement, business as usual can eventually lead to significant environmental pollution. To combat this drawback, different studies were done to develop sustainable construction materials. Microbes such as bacteria and fungi play a great role in the application of sustainable construction.

Direct and indirect application of bacteria in the construction sector is the one which most researchers argue is the most sustainable. Biocement, bioblock, and bioconcrete as self-healing, bioplastic, and biopolymer are the most well-known microbe-based construction materials. Compared to conventional concrete, bacterial concrete increases 35.15% in compressive strength, 24.32% in average tensile strength, and 17.24% in average flexural strength. And it is 4 times lower in water absorption and 8 times lower in acid resistivity than conversional concrete.

## Figures and Tables

**Table 1:** Specific microorganisms applied in the production of construction materials.

	Species name	Kingdom	Products	References
1	*Bacillus pasteurii*	Bacteria	Bioconcrete	[17, 26]
2	*Bacillus pseudifirmus*		Bioconcrete	[27]
3	*Bacillus halodurans*		Bioconcrete	[27]
4	*Bacillus cohnii*		Bioconcrete	[9]
5	*Bacillus cereus*		Bioconcrete	[21]
6	*Bacillus alkalinitrilicus*		Biocement	[28]
7	*Bacillus licheniformis*		Biocement	[28]
8	*Sphingomonas elodea*		Biopolymer	[29]
10	*Bacillus sphaericus*		Bioconcrete	[13]
11	*Bacillus lentus*		Biocement	[28]
12	*Bacillus megaterium*		Bioconcrete	[21]
13	*Bacillus sphaerius*		Bioconcrete	[17]
14	*Diaphorobacter nitroreducens*		Bioconcrete	[13]
15	*Bacillus subtilis*		Bioconcrete	[9, 16]
16	*Bacillus massiliensis*		Bioconcrete	[18]
17	*Escherichia Coli*		Bioconcrete	[17]
18	*Xanthomonas campestris*		Biopolymer	[29]
19	*Trichoderma reesei*	Fungi	Bioconcrete	[30]
20	*Oxyporus latermarginatus*		Bioblock	[23]
21	*Pleurotus salmoneo-stramineus*		Bioblock	[31]
22	*Trametes versicolor*		Bioblock	[23]
23	*Piptoporus betulinus*		Bioblock	[23]
24	*Pleurotus pulmonarius*		Bioblock	[31]
25	*Aaegerita agrocibe*		Bioblock	[31]
26	*Pleurotus ostreatus*		Bioblock	[23, 31]
27	*Ganoderma tsugae*		Bioblock	[23]
28	*Ganoderma lucidum*		Bioblock	[23, 32]
29	*Ganoderma oregonense*		Bioblock	[23]
30	*Rhizopus oryzae, Phanerochaete Chrysosporium, A. terreus, and A. oryzae, Saccharomyces cerevisiae*		Bioconcrete	[25]
31	*Aspergillus nidulans*		Bioconcrete	[33]
32	*Trichoderma reesei*		Bioconcrete	[30]

**Table 2:** Compressive, tensile, and flexural strength of bacterial and conventional concrete in 7, 14, and 28 curing days with 10^5^ cell/ml cell concentration [37].

Strength test in Mpa	Number of curing days					
	Bacterial concrete			Conventional concrete		
	7 days	14 days	28 days	7 days	14 days	28 days
Compressive strength	14.89	16.42	19.26	10.03	11.38	12.49
Tensile strength	5.33	7.28	9.36	4.69	5.19	6.34
Flexural strength	6.74	7.37	10.33	5.41	6.44	8.47

## Data Availability

All data presented or analyzed during this study are included within this article.
